# The role of immune checkpoint molecules in cancers

**DOI:** 10.3389/fimmu.2025.1674818

**Published:** 2026-01-14

**Authors:** Meilan Zhang, Junjie Xu, Zhaokuan Zheng, Zhiyun Guo, Hao Wang, Mingqing Zhou, Hailin Tang, Lewei Zhu

**Affiliations:** 1 The Affiliated Panyu Central Hospital of Guangzhou Medical University, Guangzhou, China; 2 Sun Yat-sen University Cancer Center (SYSUCC), Guangzhou, China; 3 Huadu District People’s Hospital of Guangzhou, Guangzhou, China; 4 The First People's Hospital of Foshan (The Affiliated Foshan Hospital of Southern University of Science and Technology), School of Medicine, Southern University of Science and Technology, Foshan, Guangdong, China

**Keywords:** immune checkpoint, T cell exhaustion, hematological malignancy, cancer, tumor microenvironment

## Abstract

Immune checkpoint molecules play a central role in regulating T cell function, maintaining immune homeostasis, and facilitating tumor immune evasion, making them critical targets in cancer immunotherapy. This review provides a comprehensive overview of the structural characteristics and signaling mechanisms of key co-inhibitory and co-stimulatory molecules, and their immunoregulatory roles in both solid tumors and hematological malignancies. Recent advances in the clinical application of immune checkpoint inhibitors, combination therapy strategies, and mechanisms of resistance are discussed. Furthermore, the importance of multi-target combinatorial approaches and personalized immune modulation is emphasized, offering valuable insights and directions for optimizing cancer immunotherapy strategies.

## 1 Introduction

The global incidence and mortality of cancer have been rising steadily. In 2022, there were about 20 million new cases worldwide. Nearly 9.7 million people succumbed to the disease (1, 2). Cancer is a major public health challenge. It progresses rapidly, shows high malignancy, is difficult to detect early, and has strong metastatic potential (3). Gaining a deeper understanding of the mechanisms underlying cancer development and enhancing existing therapies are critical for improving global cancer outcomes.

In recent years, immunotherapy has emerged as a central strategy in cancer treatment. It often complements surgery, chemotherapy, or radiotherapy. Its main mechanism engage and activate the immune system to recognize and eliminate tumor cells. Immunotherapies are generally classified into two types based on their modes of action: passive immunotherapy and active immunotherapy. Passive immunotherapy relies on externally produced monoclonal antibodies to target cancer cells, enhance immune responses, or inhibit tumor growth factors, thereby limiting tumor progression. In contrast, active immunotherapy stimulates the patient’s own immune system to attack cancer cells, for example, through vaccination or chimeric antigen receptor T cell therapy (4, 5).

With the continued advancement of immunotherapy, the discovery and development of immune checkpoint molecules have significantly improved treatment outcomes. In recent years, several checkpoint proteins have emerged as critical targets for immunotherapy, including T cell immunoreceptor with immunoglobulin and ITIM domains(TIGIT) (6), a poliovirus receptor-like protein (7), T cell immunoglobulin and mucin-domain containing-3 (TIM-3) (8), lymphocyte activation gene-3 (LAG-3) (9), V-domain Ig suppressor of T cell activation (VISTA) (10), sialic acid-binding immunoglobulin-like lectins 15/7/9/10 (11), B and T lymphocyte attenuator, and signal regulatory protein alpha (12). Monoclonal antibodies targeting these checkpoints are being actively developed to block immunosuppressive signals, restore T cell antitumor activity, and provide novel therapeutic strategies across a range of cancers.

Meanwhile, immune checkpoint proteins such as cytotoxic T-lymphocyte-associated protein 4 (CTLA-4), programmed cell death protein 1 (PD-1), and its ligand programmed death-ligand 1 (PD-L1) remain focal points in the field of immunotherapy research (13, 14). In common solid tumors such as lung cancer (LC) (15), melanoma (MM) (16), and breast cancer (BC) (17), aberrant activation of the PD-1/PD-L1 and CTLA-4 pathways is a key mechanism for tumor immune evasion (18, 19). These checkpoint molecules regulate T cell activity, impairing their antitumor function and thereby promoting tumor initiation and progression (20). Immune checkpoints also play critical roles in hematologic malignancies, including non-Hodgkin lymphoma and leukemia (21, 22). These types of tumors often suppress antitumor immune responses by reshaping the immune microenvironment and altering the proportion and function of immune cells, thereby promoting tumor cell survival and dissemination. For example, in certain hematologic malignancies, the PD-1/PD-L1 pathway is aberrantly activated, helping tumor cells evade recognition and elimination by the host immune system (23). Therefore, understanding the regulatory mechanisms of immune checkpoint molecules is essential not only for solid tumor therapy but also for identifying novel targets and strategies for immunologic interventions in hematologic malignancies.

With the continued advancement of multi-omics technologies, single-cell sequencing, spatial transcriptomics, and artificial intelligence, the functional landscape of the tumor immune microenvironment in solid tumors and hematologic malignancies is being explored in a more systematic and in-depth manner (24, 25). Increasing evidence indicates that immune checkpoints such as PD-1, PD-L1, CTLA-4, TIM-3, and LAG-3 not only regulate T-cell activation, exhaustion, and immune evasion, but are also closely linked to the metabolic state, inflammatory signaling, epigenetic regulation, and spatial organization of immune cells within the tumor microenvironment (26, 27). A comprehensive understanding of these pathways is reshaping our knowledge of tumorigenesis and tumor progression, while fostering the identification of novel immunoregulatory targets, combination strategies, and predictive biomarkers, thereby laying an important foundation for precision immunomodulation in both solid tumors and hematologic malignancies.

This review aims to provide an in-depth exploration of the roles of immune checkpoint molecules in cancer and hematologic malignancies, with a particular focus on their functions and mechanisms in tumor immunotherapy. It discusses the clinical efficacy of immune checkpoint inhibitors and the challenges they face, while also outlining future research directions in immune checkpoint-based therapies. By offering a comprehensive analysis of immune checkpoint molecules, this review seeks to provide both theoretical support and practical guidance for the continued advancement of cancer immunotherapy.

## 2 Mechanisms of action of immune checkpoint molecules

### 2.1 Structure and classification of immune checkpoint molecules

Dysregulation of immune checkpoint pathways is a key mechanism by which tumor cells evade elimination by the immune system (13). Immune checkpoint molecules are essential regulators of the immune response, controlling how T cells recognize and respond to antigens through the T cell receptor (TCR). These molecules are generally divided into co-stimulatory and co-inhibitory types: co-stimulatory checkpoints enhance T cell activity, while co-inhibitory checkpoints suppress it, together maintaining the balance and proper function of the immune system (28).

**Figure 1:**
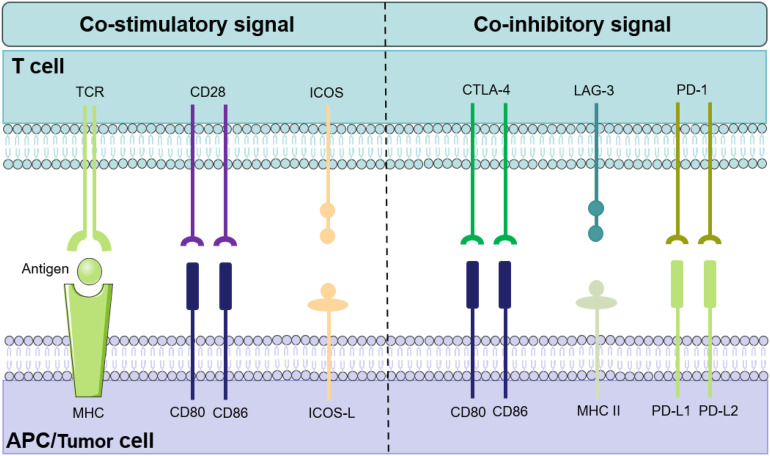
The balance between co-stimulatory and co-inhibitory signals in T cell activation. This schematic illustrates the key receptor-ligand interactions during T cell activation. The initiation of antigen-specific T cell responses depends on co-stimulatory signals, where the engagement of T cell surface receptors (e.g., CD28, ICOS) with their corresponding ligands (e.g., CD80/CD86, ICOS-L) on antigen-presenting cells effectively promotes T cell activation. In contrast, co-inhibitory signals generated by the binding of immune checkpoint molecules (e.g., CTLA-4, PD-1, LAG-3) to their ligands function as “brakes” that negatively regulate T cell function. Tumor cells exploit these co-inhibitory pathways to achieve immune evasion, and blocking co-inhibitory checkpoints has become a cornerstone strategy in cancer immunotherapy.

Co-stimulatory molecules enhance T cell activity by delivering “activation signals,” significantly increasing the efficiency of TCR signaling and thereby lowering the threshold for TCR recognition of the MHC-peptide complex (29). For example, B7 family proteins (such as B7-1/CD80 and B7-2/CD86) on the surface of antigen-presenting cells (APCs) can bind to receptors on T cells and participate in the regulation of T cell immune activity (30). Binding of B7 proteins to CD28 activates T cells, promoting their proliferation and differentiation, upregulating IL-2 receptor expression, and enhancing IL-2 production, thereby strengthening the immune response (31). In contrast, when B7 proteins bind to CTLA-4, they deliver co-inhibitory signals that suppress T cell activation. CTLA-4 competes with CD28 for binding to B7 proteins, thereby blocking CD28-mediated positive signaling. Moreover, CTLA-4 can induce T cell apoptosis, inhibit the production of IL-2, and downregulate the expression of IL-2 receptors, further limiting T cell immune function (32). Therefore, the dynamic balance between co-stimulatory and co-inhibitory signals is critical for maintaining immune homeostasis. Tumor cells often disrupt this balance by attenuating co-stimulatory pathways or enhancing inhibitory signals to achieve immune evasion (**Figure 1**).

### 2.2 Co-stimulatory molecules

Co-stimulatory signals are essential for effective T cell activation and are finely balanced by co-inhibitory pathways. Structurally, these molecules mainly belong to the immunoglobulin superfamily or the tumor necrosis factor receptor superfamily. Activating checkpoint molecules enhance antitumor immunity by promoting T cell activation, proliferation, and cytokine production. For instance, 4-1BB and CD28, which are upregulated upon T cell activation, can strengthen T cell function and maintain their effector state through ligand engagement. These co-stimulatory pathways are increasingly recognized as promising targets for cancer immunotherapy.

#### 2.2.1 CD28

Effective activation of T cells relies on the synergistic action of two signals: the first is the specific recognition of the MHC–antigen peptide complex by the TCR, and the second is mediated by the interaction between CD28 and its B7 ligands (33–35). CD28-mediated co-stimulatory signaling activates key pathways such as PI3K/Akt and NF-κB, amplifies TCR signaling, and promotes IL-2 production. Additionally, CD28 regulates the expression of enzymes involved in glucose metabolism, drives T cell metabolic reprogramming, and upregulates the anti-apoptotic protein Bcl-xL to support cell survival (36, 37). Absence of CD28 signaling can lead to T cell dysfunction or programmed cell death (38). As a key regulatory molecule in the T cell co-stimulatory signaling pathway, CD28 plays a critical role in T cell activation, making it an important research target and therapeutic (39).

#### 2.2.2 Inducible T-cell co-stimulator

ICOS is homologous to the co-stimulatory receptor CD28 and the co-inhibitory receptor CTLA-4. Unlike the constitutive activation of CD28, ICOS signaling depends on inducible expression following T cell activation and is considered a “booster” of T cell responses, mainly involved in sustaining and optimizing immune responses during the later stages (40). ICOS does not participate directly in the initial phase of T cell activation but plays a critical regulatory role by activating key signaling pathways such as PI3K-Akt-mTOR, NFAT, and MAPK, thereby modulating the differentiation, function, and survival of follicular helper T cells (Tfh) (41). Within the tumor microenvironment, ICOS exhibits bidirectional regulatory properties: on one hand, it can enhance effector T cell-mediated antitumor immune responses; on the other hand, it may promote the immunosuppressive function of regulatory T cells, thereby influencing the overall efficacy of immunotherapy (42). Studies have shown that ICOS expression patterns and their association with clinical outcomes vary across different tumor types, and their function may be influenced by the immune context. In colorectal cancer, high ICOS expression has been associated with increased enrichment of Foxp3^+^ tumor-infiltrating lymphocytes (TILs), potentially driving the progression from precancerous lesions to malignancy (43). Conversely, other studies have demonstrated that high ICOS expression in Th1-type CD4^+^ T cells is correlated with prolonged patient survival (44). These findings suggest that the role of ICOS may be heterogeneous across different immune cell subsets and could exert dual effects on tumor progression and prognosis. Therapies targeting ICOS face challenges including microenvironmental heterogeneity, functional conflicts among T cell subsets, and adaptive resistance. Future strategies may require spatiotemporal-specific regulation and combination approaches targeting multiple pathways to achieve precise immune remodeling and enhance the efficacy of antitumor immunotherapy.

#### 2.2.3 Tumor necrosis factor receptor superfamily member 9

4-1BB (CD137) is a costimulatory receptor primarily expressed on activated CD4^+^ and CD8^+^ T cells, NK cells, dendritic cells (DCs), and certain myeloid cell subsets. Upon binding to its ligand 4-1BBL, this receptor promotes T cell survival, proliferation, and antitumor activity (45). The 4-1BB/4-1BBL interaction triggers the recruitment of TRAF1/TRAF2 complexes and the E3 ubiquitin ligases cIAP1/2, subsequently activating key signaling cascades, including NF-κB, ERK, p38 MAPK, and JNK pathways. These cascades synergistically amplify co-stimulatory signals, enhancing the differentiation, expansion, and effector functions of both CD4^+^ and CD8^+^ T cells (46, 47).

Inhibitory immune checkpoints are key regulators of tumor immune evasion. Their expression is upregulated during sustained T cell activation, delivering negative signals that suppress T cell activation, proliferation, and cytotoxicity, thereby weakening antitumor immune responses. Under physiological conditions, these molecules maintain immune homeostasis and prevent excessive immune activation and autoimmunity. However, in the tumor microenvironment, their persistent overexpression leads to T cell exhaustion and immune tolerance, ultimately impairing tumor clearance. Well-characterized inhibitory checkpoints include PD-1, PD-L1, and CTLA-4, while emerging targets such as LAG-3 and TIM-3 have recently attracted increasing attention.

### 2.3 Co-inhibitory molecules

#### 2.3.1 CTLA-4

**Figure 2:**
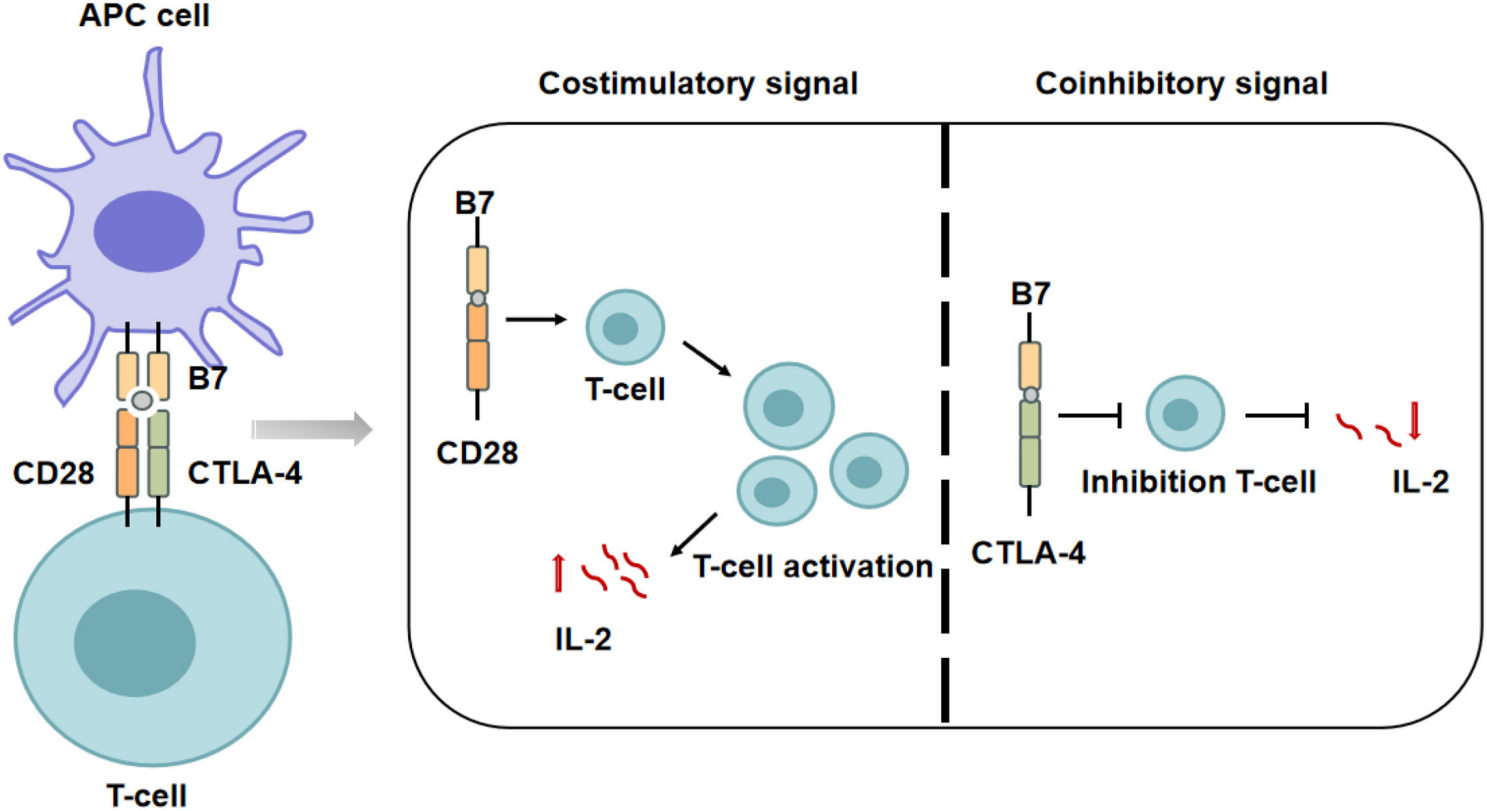
Regulation of T cell activation by competitive binding of CTLA-4 and CD28 to B7 molecules. B7 family proteins expressed on the surface of APCs can bind to receptors on the surface of T cells and transmit distinct signals, thereby enabling precise regulation of T cell functions. When B7 proteins bind to CD28, they deliver a costimulatory signal that activates T cells, induces their proliferation and differentiation, upregulates IL-2 receptor expression, promotes the synthesis and secretion of IL-2, and consequently enhances the strength of the immune response. In contrast, the binding of B7 proteins to CTLA-4 transmits a coinhibitory signal that suppresses T cell activation.

CTLA-4, also known as CD152, is a member of the immunoglobulin superfamily and functions as a key inhibitory immune checkpoint. It is primarily expressed on regulatory T cells, as well as activated CD4^+^ and CD8^+^ T cells. CTLA-4 is mainly stored in intracellular vesicles and translocated to the cell surface upon TCR activation, a process mediated by T cell receptor–interacting molecule (TRIM) and maintained through phosphorylation-dependent mechanisms (48). CTLA-4 shares the ligands B7-1 and B7-2 with CD28 but has a significantly higher affinity for both, allowing it to outcompete CD28 for binding and thereby block CD28-mediated costimulatory signaling. This competitive binding effectively suppresses T cell activation and proliferation (49). In addition to ligand competition, CTLA-4 can mediate trans-endocytosis of CD80/CD86 from the surface of APCs, thereby reducing the availability of costimulatory molecules at the immunological synapse and further enhancing its immunosuppressive function (50) (**Figure 2**).

#### 2.3.2 PD-1/PD-L1

PD-1 is primarily expressed on activated T cells, B cells, and certain subsets of myeloid cells (51). In tumor-specific T cells, PD-1 expression is markedly upregulated. It is transcriptionally regulated by multiple transcription factors, including nuclear factor of activated T cells, the NOTCH signaling pathway, forkhead box O1, and interferon regulatory factor 9 (52, 53). In naive CD8^+^ T cells, which do not express PD-1, conserved regions B and C of the PDCD1 gene are highly methylated, thereby repressing its transcriptional activity (54). In contrast, during chronic infections or within the tumor microenvironment, the promoter region of the Programmed Cell Death 1(PDCD1) gene undergoes demethylation, facilitating the sustained expression of PD-1 in exhausted CD8^+^ T cells (55). Additionally, IFN-α can activate IRF9 via the JAK-STAT signaling pathway. As part of the ISGF3 complex, IRF9 binds to interferon-stimulated response elements in the PDCD1 promoter, further enhancing PD-1 transcription (56). Signals released by tumor cells have also been shown to upregulate the expression of c-FOS, which indirectly promotes PD-1 expression in tumor-infiltrating T cells (57). Functionally, PD-1 plays a dual role. It prevents excessive immune activation and maintains peripheral tolerance, protecting against autoimmunity, but its persistent expression in the tumor microenvironment suppresses T cell–mediated antitumor responses, facilitating immune evasion. The major ligand, PD-L1, is broadly expressed on antigen-presenting cells and tumor cells and is strongly induced by inflammatory cytokines such as IFN-γ. Upon binding, PD-1 transduces inhibitory signals through its intracellular immunoreceptor tyrosine-based inhibitory motif (ITIM) and switch motif (ITSM), recruiting phosphatases such as SHP-2. This inhibits downstream TCR and CD28 signaling, ultimately restraining T cell activation, proliferation, and effector function (58).

Within the tumor microenvironment, the interaction between PD-L1 on tumor cells and PD-1 on T cells disrupts TCR and costimulatory signaling, leading to effector T cell dysfunction and exhaustion, thereby facilitating tumor immune evasion (59, 60). PD-L1 expression is closely associated with CD8^+^ T cell infiltration, Th1-type cytokines, chemokines, interferon levels, and specific immune-related gene signatures (61). Beyond its classical immunosuppressive role, PD-L1 can also bind to CD80 on activated T cells, further dampening T cell responses and inducing apoptosis (62). Studies have shown that, under the combined stimulation of tumor antigens and inflammatory cytokines, tumor-infiltrating T cells themselves can also express PD-L1. This PD-L1 expression mediates reverse inhibitory signaling, further contributing to the immunosuppressive tumor microenvironment (63). In addition to its role in immune evasion, PD-L1 also possesses tumor-intrinsic pro-oncogenic functions. It can activate intracellular signaling pathways that enhance cell proliferation and survival, induce epithelial–mesenchymal transition (EMT), and promote the acquisition of stem cell-like phenotypes, thereby accelerating tumor progression (64). Collectively, PD-L1 plays a critical role in both immune suppression and malignant transformation, making it one of the most important targets in cancer immunotherapy.

#### 2.3.3 LAG-3

LAG-3 is a transmembrane protein predominantly expressed on activated CD4^+^ and CD8^+^ T cells, regulatory T cells (Tregs), and natural killer (NK) cells (26). Its gene is located on human chromosome 12 and encodes a protein structurally similar to CD4, though it primarily exerts immunosuppressive functions (65). LAG-3 binds to major histocompatibility complex class II molecules with high affinity, thereby blocking TCR signaling, inhibiting T cell activation and proliferation, and reducing cytokine production, ultimately contributing to T cell exhaustion (66). High LAG-3 expression is frequently associated with poor clinical outcomes, particularly through its role in promoting tumor immune evasion, enhancing Treg function, and shaping an immunosuppressive tumor microenvironment, as observed in cancers such as NSCLC, colorectal cancer, and head and neck squamous cell carcinoma. Interestingly, in certain malignancies, including breast and gastric cancers, LAG-3 single positivity or co-expression with PD-1 has sometimes been linked to improved survival outcomes. This suggests that, in specific patient subsets—particularly those with favorable responses to therapy—LAG-3 expression may reflect an active antitumor immune response (67–69). Therapeutically, LAG-3 blockade combined with PD-1 or PD-L1 inhibitors has shown synergistic immunostimulatory effects, suppressing Treg-mediated immunosuppression, promoting dendritic cell maturation, and restoring the activity of exhausted CD4^+^ and CD8^+^ T cells within the tumor microenvironment, thereby enhancing antitumor immunity (70–73).

#### 2.3.4 TIM-3

TIM-3, also known as hepatitis A virus cellular receptor 2, is a critical immune checkpoint molecule first identified in 2001 through studies on asthma susceptibility genes in mice (74). Structurally, TIM-3 comprises an N-terminal immunoglobulin variable domain, a mucin-like domain, a transmembrane region, and a cytoplasmic tail. The cytoplasmic region contains multiple tyrosine residues, which can be phosphorylated by kinases such as Itk, Fyn, and Lck upon ligand binding, thereby activating downstream signaling pathways including PI3K, PLC-γ1, NFAT, and NF-κB (75–77). In addition to its membrane-bound form, a soluble splice variant of TIM-3 has been identified, which can competitively bind to TIM-3 ligands and modulate immune responses by acting as a decoy receptor (78–80). TIM-3 primarily engages ligands such as Galectin-9 and CEACAM-1 to deliver inhibitory signals, limiting excessive immune activation and maintaining tolerance (81). It is broadly expressed on various immune cells, including T cells, NK cells, macrophages, and DCS (82). TIM-3 expression has been detected in a variety of malignancies, including MM, acute myeloid leukemia (AML), NSCLC, prostate cancer, osteosarcoma, colorectal cancer, and hepatocellular carcinoma (HCC) (26). Within the tumor microenvironment, elevated TIM-3 levels contribute to T cell exhaustion, enhance immune suppression, and facilitate immune evasion. Co-expression of TIM-3 and PD-1 on TIL shas been shown to synergistically impair T cell function, highlighting TIM-3 as a promising therapeutic target in cancer immunotherapy.

#### 2.3.5 TIGIT

TIGIT, an inhibitory receptor expressed on NK cells, T cells, and DCs, can promote tumor immune evasion through multiple mechanisms. First, it competitively binds to CD155/CD112 expressed on tumor cells, thereby weakening CD226-mediated costimulatory signals and reducing the cytotoxic activity of NK cells and T cells (83). In addition, high TIGIT expression in Tregs can enhance immunosuppressive effects by modulating DC activity through the secretion of IL-10 and TGF-β (84), while also promoting the polarization of tumor-associated macrophages toward the M2 phenotype (85, 86). Upregulation of TIGIT is also closely associated with T cell exhaustion; it synergizes with PD-1 to inhibit CD226-mediated costimulation and PI3K signaling, ultimately leading to functional impairment of T cells (46). As summarized in **Table 1**, a spectrum of immune checkpoint-targeted therapies have now entered clinical investigation.

**Table 1:** Clinical trials of immune checkpoint-targeting therapies.

Immune checkpoint	Drugs	NCT	Cancer type
4- lBB (CD137)	Urelumab	NCT02 l l0082	Advanced/metastatic solid tumors
CD47	Magrolimab + Azacitidine	NCT043 l388l	High-risk MDS *I* AML
CD47	Magrolimab (early)	NCT03248479	AML / MDS
CD47	Magrolimab (other)	NCT0443569 l	AML / MDS
ICOS	Vopratelimab (JTX-2011)	NCT03989362	Advanced solid tumors
LAG-3 + PD-1	Relatlimab + Nivolumab	NCT03470922	Advanced melanoma
PD-1	Pembrolizumab	NCT02142738	NSCLC (PD-Ll 2:50%)
PD-Ll TIGIT	Atezolizumab + nab-paclitaxel Vibostolimab (MK-7684)	NCT0242589 l NCT02964013	Metastatic TNBC Various solid tumors *I* NSCLC
TIGIT + PD-Ll	Tiragolumab + Atezolizumab	NCT03563716	NSCLC (PD-Ll high)
TIM-3	Sabatolimab (MBG453) + HMA	NCT04878432	High-risk MDS (with azacitidine)
TIM-3	Sabatolimab	NCT04623216	AML (MRD+ post HSCT)

All data were sourced and verified from ClinicalTrials.gov. The NCT number for each trial is hyperlinked to its official page for detailed information.

## 3 Research and immunotherapeutic advances of co-stimulatory molecules in cancer

### 3.1 Solid tumors

Co-stimulatory molecules play a pivotal role in T cell activation and are essential for initiating effective antigen-specific immune responses and establishing long-term immune memory. According to the classical two-signal model, engagement of the TCR alone is insufficient to fully activate T cells; co-stimulatory signals are required to promote clonal expansion and effector differentiation. In solid tumors, malignant cells can disrupt the formation of the immunological synapse by downregulating co-stimulatory ligands and upregulating inhibitory molecules, thereby suppressing T cell activity, inducing immune tolerance, and facilitating immune evasion. To overcome these immunosuppressive mechanisms, immunostimulatory agents targeting co-stimulatory pathways have emerged as promising therapeutic strategies. Activation of molecules such as CD28, ICOS, and 4-1BB has been shown to restore the function of T cells and NK cells and to improve the immunosuppressive tumor microenvironment. Several agents targeting these pathways have demonstrated the potential to enhance antitumor immune responses in preclinical studies and early-phase clinical trials.

#### 3.1.1 CD28

Optimization of CD28 signaling motifs and CAR constructs holds promise for improving therapeutic outcomes in solid tumors while minimizing toxicity (87). In addition, CD28 has demonstrated significant advantages in CAR-NK cell platforms. Studies have shown that CD28-based CAR-NK cells exhibit stronger activation, cytotoxicity, and antitumor effects compared to those incorporating 4-1BB. This enhanced activity may be attributed to the superior ability of CD28 to recruit ZAP70 and regulate key downstream signaling molecules such as MAP3K8, suggesting its important therapeutic potential in optimizing CAR-NK cell–based immunotherapy for solid tumors (88).

In addition, CD28 plays an important role in modulating responses to both chemotherapy and immunotherapy. In patients with triple-negative breast cancer (TNBC), significant changes in CD28^+^ T cell levels were observed before and after neoadjuvant chemotherapy, suggesting that CD28^+^ T cells may be involved in chemotherapy-induced immune modulation and associated with treatment sensitivity (89). In non-small cell lung cancer (NSCLC), although immune checkpoint blockade (ICB) has improved patient survival, resistance remains a major clinical challenge. Recent studies have identified that tumor-infiltrating PD-1^+^CD28^+^ T cells retain multifunctional characteristics and are strongly associated with favorable responses to ICB therapy, whereas PD-1^+^CD28^-^ T cells display an exhausted phenotype (90). Similarly, in patients with metastatic colorectal cancer treated with bevacizumab combined with chemotherapy, higher levels of peripheral CD8^+^CD28^+^ T cells were positively correlated with better therapeutic outcomes (91). Of particular interest, a bispecific antibody targeting bo th CD28 and Nectin-4 has been developed to enhance T cell co-stimulation within the tumor microenvironment, significantly boosting T cell activation and cytotoxicity. This strategy offers a novel immunotherapeutic approach for Nectin-4–positive solid tumors, including urothelial carcinoma (92). The trispecific antibody TsAb-B7-H4, which simultaneously targets B7-H4/CD3/CD28, markedly enhances cytotoxic activity against CRC without inducing inflammatory cytokine release, providing a promising new immunotherapeutic strategy for CRC (93). The clinical advancement of CD28-targeted immunotherapy depends on simultaneously evaluating its antitumor efficacy, durability of immune responses, and controllable toxicity, as well as systematically assessing various combinatorial strategies and immune cell states to identify the most effective and safest therapeutic approach (39).

#### 3.1.2 ICOS

Clinical research on ICOS agonists has primarily focused on two antibodies: vopratelimab (JTX-2011, IgG1κ) and feladilimab (GSK3359609, IgG4-PE variant). Vopratelimab requires antigen-induced T cell activation and subsequent upregulation of ICOS for effective binding, thereby promoting CD4^+^ T cell proliferation. In the ICONIC trial (Phase I/II, evaluating vopratelimab as monotherapy or in combination with nivolumab in refractory solid tumors), vopratelimab demonstrated good tolerability, with a recommended dose of 0.3 mg/kg every three weeks. However, the objective response rate (ORR) for both monotherapy and combination therapy were relatively low, at approximately 1.4% and 2.3%, respectively, with most patients failing to exhibit sustained anti-tumor responses (94). Feladilimab (GSK3359609), in combination with pembrolizumab, was evaluated in patients with head and neck squamous cell carcinoma. Still, the trial was terminated early due to failure to meet the expected survival benefit (95). In addition to direct ICOS agonist antibodies, various combination strategies targeting the ICOS pathway have shown encouraging progress. Preclinical studies indicate that ICOS agonists, whether administered alone or in combination with anti–PD-1 antibodies, can enhance CD8^+^ T cell infiltration in both tumors and draining lymph nodes, thereby activating anti-tumor immune responses (96). Moreover, other studies have demonstrated that combining ICOS agonists with CTLA-4 inhibitors significantly augments anti-tumor immunity, providing a strong rationale for further clinical investigation (97). The application of ICOS in chimeric antigen receptor T-cell (CAR-T) therapy has also garnered increasing attention. Incorporation of a constitutively active ICOS co-stimulatory signal into B7H3-CAR-T cells significantly enhances anti-tumor activity against TNBC. This enhancement includes increased T cell proliferation, cytokine secretion, and cytotoxicity, demonstrating superior therapeutic efficacy compared to conventional B7H3-CAR-T cells in xenograft mouse models (98). Furthermore, complement regulatory factor H has been identified as an ICOS ligand capable of supporting regulatory T cell survival and function within the glioma microenvironment, thereby promoting an immunosuppressive state (99). ICOS-modified B7H3-CAR-T cells enhance cytokine secretion and cytotoxicity through ICOS signaling, exhibiting superior antitumor activity and prolonged survival compared with conventional B7H3-CAR-T cells in TNBC models (98).

#### 3.1.3 4-1BB(CD137)

4-1BB is an important co-stimulatory receptor in cancer immunotherapy. Upon binding to its ligand, it enhances T cell effector functions, including promoting the secretion of IFN-γ and cytotoxic molecules, increasing the proliferation and survival of CD8^+^ T cells, and inhibiting their apoptosis. *In vivo*, anti–4-1BB antibodies mediate anti-tumor effects primarily through the activation of CD8^+^ T cells (91). Two 4-1BB agonist antibodies, Urelumab (BMS-663513, IgG4) and Utomilumab (PF-05082566, IgG2), have been extensively studied. Urelumab showed measurable antitumor activity as monotherapy in early trials, but its development was limited by dose-dependent hepatotoxicity. At doses ≥1.0 mg/kg, severe transaminase elevations occurred, with some fatal outcomes. Subsequent studies established 0.1 mg/kg every three weeks as a safer dose, where the most common adverse events were mild fatigue and nausea (92).

To enhance therapeutic efficacy, 4-1BB–based bispecific antibodies have emerged as a research focus in recent years. For example, the TIGIT×4-1BB bispecific antibody ABL112 can enhance anti-tumor immune responses by activating 4-1BB–mediated co-stimulatory signaling as well as FcγR-dependent macrophage activation (100). Another bispecific antibody, givastomig (ABL111), which targets both CLDN18.2 and 4-1BB, selectively activates 4-1BB^+^ T cells within the tumor microenvironment, thereby enhancing local immune responses while minimizing systemic immune activation and associated toxicities (101). In addition, the combination of the OX40 agonist antibody Ivuxolimab with the 4-1BB agonist antibody Utomilumab has demonstrated synergistic activation of immune pathways. This dual-targeted strategy has shown preliminary anti-tumor activity and favorable tolerability in patients with various advanced solid tumors (102).

The role of 4-1BB in regulating tumor immune tolerance has also garnered increasing attention. In HCC, anti–PD-1 therapy may promote the migration and expansion of Tregs, leading to elevated intratumoral Treg levels. Studies have shown that Nrp-1 and 4-1BB cooperate in this process, suggesting that the combination of Nrp-1 inhibitors with 4-1BB agonists may enhance the anti-tumor effects mediated by PD-1 blockade (103). In pancreatic cancer, the frequency of tumor-associated antigen-specific IFN-γ^+^4-1BB^+^ CD8^+^ T cells in peripheral blood is closely associated with treatment response, indicating their potential as predictive biomarkers for the efficacy of immunotherapy and neoadjuvant chemotherapy (104). Furthermore, in patients with recurrent or metastatic head and neck squamous cell carcinoma, a circulating CD137^+^ T cell proportion ≥1.65% has been correlated with significantly prolonged progression-free survival (PFS) and overall survival (OS), suggesting that CD137^+^ T cells may also serve as a potential prognostic biomarker (105).

### 3.2 Hematologic malignancies

In hematologic malignancies, the introduction of PD-1–CD28 chimeric switch receptors has been shown to significantly enhance the expansion and anti-tumor efficacy of tumor-infiltrating lymphocytes. The CD28 co-stimulatory signal helps T cells overcome the immunosuppressive effects of the PD-1/PD-L1 axis, thereby strengthening the immune response (106). Studies have demonstrated that although PD-1^+^ T cells in peripheral blood possess potent cytotoxic potential, their proliferative capacity remains limited. By introducing a PD-1/CD28 fusion receptor in combination with CD19-directed CAR, the proliferative ability and anti-tumor activity of these T cells can be markedly improved (107). The anti-ICOS antibody MEDI-570 has shown preliminary efficacy in angioimmunoblastic T-cell lymphoma, suggesting that ICOS-targeted strategies hold promise in the treatment of hematologic malignancies (108). Additionally, combination therapy using Utomilumab and an anti-CD20 monoclonal antibody showed enhanced clinical benefit in B-cell lymphomas, including follicular lymphoma. In a dose-expansion cohort of 48 patients, the overall response rate (ORR) reached 21.2%, with four patients achieving complete response. The combination was generally well tolerated, with no dose-limiting toxicities and low immunogenicity (109).

## 4 Research and immunotherapeutic advances of co-inhibitory molecules in cancer

### 4.1 Solid Tumors

#### 4.1.1 CTLA-4

CTLA-4 inhibitors have become foundational in cancer immunotherapy by enhancing T cell function through blockade of inhibitory signaling. Ipilimumab, the first FDA-approved CTLA-4 inhibitor, is a fully human IgG1κ monoclonal antibody that prevents CTLA-4 from binding its ligands, thereby boosting T cell activity. In 2011, ipilimumab was approved for metastatic melanoma, and a Phase III trial demonstrated a significant improvement in median overall survival (OS) from 6.4 to 10.0 months, establishing a clear survival benefit (110). Tremelimumab is another humanized IgG2 monoclonal antibody targeting CTLA-4, with a mechanism of action similar to that of ipilimumab. In the POSEIDON study, the combination of tremelimumab with durvalumab and chemotherapy was evaluated as first-line treatment for patients with advanced NSCLC. The results showed that this combination significantly improved both median OS and PFS (111).

Next-generation CTLA-4 antibodies and bispecific antibodies are gradually achieving more precise targeting and hold promise for reducing the risk of immune-related toxicities. For instance, 2MW4691 is a bispecific antibody targeting both CCR8 and CTLA-4, capable of selectively depleting tumor-infiltrating Treg cells via ADCC mechanisms, thereby enhancing the anti-tumor activity of CD8^+^ T cells and demonstrating favorable safety and therapeutic potential (112). XTX101 employs a masked peptide design that allows activation only within the tumor microenvironment by specific proteases. It also enhances affinity for FcγRIII to mediate Treg depletion, showing improved anti-tumor efficacy and significantly reduced peripheral immune toxicity in both animal and ex vivo models (113). In addition, Fc-enhanced CTLA-4 antibodies (FcE-aCTLA-4) efficiently bind to FcγRIV expressed by tumor-associated macrophages, selectively depleting intratumoral Treg cells. When combined with doxorubicin, anti–PD–1 antibodies, and sonoporation-assisted LIPU/MB therapy, this antibody induced a cure rate of up to 90% in resistant glioblastoma models and established durable immune memory, indicating remarkable anti-tumor potential (114). Moreover, CTLA-4 nanobodies have also emerged as a research hotspot. A nanobody developed by Iranian researchers significantly suppressed tumor progression in a murine MM model (115).

#### 4.1.2 PD-1/PD-L1

Monoclonal antibodies targeting PD-1 or PD-L1 have been developed to block this signaling pathway, effectively restoring T cell–mediated anti-tumor immunity. This approach has become one of the central strategies in current cancer immunotherapy. The FDA has approved several immune checkpoint inhibitors targeting the PD-1/PD-L1 pathway for clinical use. Approved PD-1 inhibitors include nivolumab, pembrolizumab, and cemiplimab (116–118). The details of FDA-approved PD-1 inhibitors are summarized in **Table 2**. while PD-L1 inhibitors include atezolizumab, durvalumab, and avelumab (119–121). In NSCLC, first-line treatment with pembrolizumab in patients with high PD-L1 expression (≥50%) significantly PFS and OS compared to conventional platinum-based chemotherapy (122). The phase III PACIFIC trial demonstrated that durvalumab increased the ORR to 66.3% in unresectable stage III NSCLC and significantly improved OS (123). In metastatic squamous NSCLC, the combination of pembrolizumab with chemotherapy further improved both PFS and OS (124). In HCC, the phase III IMbrave150 trial confirmed that atezolizumab plus bevacizumab achieved a 12-month OS rate of 67.2%, superior to sorafenib, with a reduced risk of death (125, 126). Postoperative recurrence of liver cancer has been linked to PD-L1 upregulation and CD8^+^ T cell–mediated immune pressure, suggesting potential value for PD-L1 blockade in the adjuvant setting (127). MM, known for its high sensitivity to immunotherapy, has seen nivolumab and pembrolizumab approved for metastatic disease, with monotherapy ORRs of 31.7% and 33%, respectively (128, 129). When combined with the CTLA-4 inhibitor ipilimumab, the 5-year OS rate increases to 52% (130). Furthermore, combining oncolytic viruses with PD-1 inhibitors has been shown to enhance both local and systemic anti-tumor immunity in glioblastoma (131). In TNBC, LSD1 inhibitors can specifically upregulate H3K4me2 modifications at the PD-L1 promoter, enhancing PD-L1 expression. When combined with PD-1 antibodies, a synergistic antitumor effect is observed (132). The details of FDA-approved PD-L1 inhibitors are summarized in **Table 3**.

**Table 2:** The details of the FDA-approved inhibitors of PD-1.

Target	Drugs (Brand name)	Description	Time of approval	Cancer type
PD-1	Tislelizumab (Tevimbra)	Humanized IgG4	2024	ESCC;NSCLC
	Tislelizumab (Tevimbra)	Humanized IgG4	2023	ESCC; NSCLC
	Dostarlimab (Jemperli)	Humanized IgG4	2021	EC; dMMR solid cancers
	Cemiplimab (Libtayo)	Human lgG4	2018	CSCC; NSCLC; Basal cell carcinoma;Cervical cancer
	Nivolumab (Opdivo)	Human lgG4	2014	Melanoma; NSCLC; RCC; cHL; HNSCC; UC; Gastric cancer; HCC; CRC; Malignantpleural mesothelioma; ESCC; OC
	Pembrolizumab (Keytruda)	Humanized IgG4	2014	Melanoma; NSCLC; HNSCC; cHL; Gastric cancer; Cervical cancer; HCC; MCC; CRC; ES-SCLC; cancers; TNBC; CSCC; UC; Primary mediastinal large B-cell lymphoma; ESCCRCC; EC; TMB-high solid

**Table 3:** The details of the FDA-approved inhibitors of PD-L1.

Target	Drugs (Brand name)	Description	Time of approval	Cancer type
PD-LI	Cosibelimab (UNLOXCYT)	Human lgGl	2024	cSCC
	Avelumab (Bavencio)	Humanized IgGl	2017	MCC; UC; RCC; OC
	Durvalumab (Imfinzi)	Humanized IgGl	2017	UC; NSCLC; ES-SCLC; HCC
	Atezolizumab (Tecentriq)	Human lgGl	2016	UC; NSCLC; TNBC; HCC; ES-SCLC

Combination therapy strategies have become critical for enhancing the efficacy and durability of responses to PD-1/PD-L1 inhibitors. In HCC, the combination of atezolizumab and bevacizumab has demonstrated robust synergistic anti-tumor activity (125). In advanced renal cell carcinoma, treatment with axitinib plus pembrolizumab achieved an ORR of up to 73% (133). For BRCA-mutated BC, the PARP inhibitor olaparib combined with durvalumab yielded an ORR of 52%, indicating the advantage of dual mechanistic synergy (134). In addition, radiotherapy can promote the release of tumor antigens and activate DCS; its combination with PD-1 inhibitors significantly improves response rates in non-irradiated lesions (135). Oncolytic virus–based approaches have also shown promise—for example, the oncolytic virus Ad-Cab, engineered to express a PD-L1 antagonist peptide, effectively activates neutrophils and NK cells, thereby enhancing antitumor immune responses (136).

Although PD-1/PD-L1 immunotherapy has achieved groundbreaking progress across various tumor types, its efficacy remains limited by multiple factors. A subset of patients develops hyperprogressive disease, which has been closely associated with an increased proportion of peripheral exhausted CD8^+^ T cells (137). In addition, PD-L1 can undergo nuclear translocation and activate the AKT/ERK signaling pathway via the Gas6/MerTK axis, thereby promoting tumor proliferation and inducing immune resistance (138). Lactate metabolic byproducts can also enhance PD-1 expression by modulating Treg cell metabolism, contributing to an immunosuppressive microenvironment (139). Therefore, understanding the mechanisms of resistance and developing strategies to regulate PD-L1 expression, metabolic reprogramming, and immune cell function will provide new avenues to overcome resistance and expand the population benefiting from immunotherapy.

#### 4.1.3 LAG-3

Preclinical studies have demonstrated high LAG-3 expression in various tumor models, including colorectal cancer, MM, head and neck squamous cell carcinoma, and NSCLC, particularly within tumor-infiltrating lymphocytes, where it is frequently co-expressed with PD-1, contributing to the induction and maintenance of T cell exhaustion (140–143). Functional assays further indicate that LAG-3 monoclonal antibody therapy, either alone or in combination with PD-1 inhibitors, can effectively restore T cell activity and enhance anti-tumor immune responses (144, 145). For example, dual blockade of LAG-3 and PD-1 significantly increases the expression of granzyme B and IFN-γ in CD8^+^ T cells, thereby suppressing tumor progression (144).

In clinical research, the RELATIVITY-047 trial (NCT03470922) demonstrated that the combination of the LAG-3 monoclonal antibody relatlimab with the PD-1 inhibitor nivolumab significantly prolonged PFS in patients with unresectable or metastatic MM, leading to the first FDA and EMA approval of a LAG-3–based combination immunotherapy regimen (71). Another study showed that fianlimab combined with cemiplimab also exhibited strong efficacy in advanced MM, outperforming either monotherapy (146). Phase II/III clinical trials of relatlimab plus PD-1 inhibitors are actively ongoing in colorectal cancer, gastric cancer, and bladder cancer (147–149). Notably, in MSI-H/dMMR colorectal cancer, this combination has shown higher ORR compared to PD-1 monotherapy (150, 151), suggesting that LAG-3–targeted strategies may offer greater therapeutic potential in specific molecular subtypes.

#### 4.1.4 TIM-3

In recent years, TIM-3 has emerged as a novel immune checkpoint molecule, and its immunoregulatory role in solid tumors has gained increasing attention. Studies have shown that TIM-3 is persistently overexpressed in T cells associated with various solid tumors, typically accompanied by T cell exhaustion, and is closely linked to impaired immune responses and tumor immune evasion. The combination of anti–TIM-3 antibodies with PD-1 inhibitors significantly enhances CD8^+^ T cell infiltration in tumor tissues and upregulates cytotoxic factors such as IFN-γ and granzyme B, thereby augmenting anti-tumor immune activity and prolonging survival (152, 153). In murine models of HCC, dual blockade of TIM-3 and PD-1 markedly suppresses tumor growth, reduces immunosuppressive cytokines IL-6 and IL-10, and promotes IFN-γ and TNF-α production (154). In addition to antibody-based therapies, small-molecule inhibitors targeting TIM-3 have also shown promising therapeutic potential. ML-T7 enhances the function of CD8^+^ T cells and NK cells by binding to the FG-CC′ loop of TIM-3, thereby blocking its interaction with phosphatidylserine and CEACAM-1 (155), SMI402 interferes with multiple TIM-3 ligand interactions, including those with HMGB1, PtdSer, and CEACAM-1, which improves antigen presentation, promotes T cell infiltration, and effectively inhibits tumor progression (156). Notably, TIM-3 can also be expressed by tumor cells themselves and may directly promote tumor progression. In HCC and NSCLC, TIM-3 has been shown to enhance tumor growth by activating the NF-κB/IL-6/STAT3 signaling pathway. However, in MM, TIM-3 may exert tumor-suppressive effects, suggesting that its biological function is tumor type–dependent (26, 157). Recent studies have also found that TIM-3 is highly expressed during the precancerous stage of lung adenocarcinoma, with expression gradually declining as the disease progresses. In mouse models, blocking TIM-3 during the precancerous phase significantly reduced tumor burden, improved antigen presentation, T cell activation, and the M1/M2 macrophage ratio, indicating that TIM-3 may serve as a potential target for early immunointervention in lung adenocarcinoma (158). In clinical settings, the TIM-3 monoclonal antibody LY3321367, either as monotherapy or in combination with a PD-L1 antibody, has demonstrated favorable safety and pharmacokinetic profiles in the treatment of advanced solid tumors. Although its overall anti-tumor activity was limited, increased CD8^+^ T cell infiltration was observed in some patients, suggesting a potential for immune activation (159). Additionally, TIM-3 aptamers, as non-antibody therapeutic agents, have also shown promise. In a murine model of diffuse midline glioma, TIM-3 aptamers promoted immune cell infiltration and activated tumor-specific immune responses, thereby extending survival duration (160).

#### 4.1.5 TIGIT

Numerous studies have established TIGIT as a critical target in cancer immunotherapy. In gastric cancer, PRDM15 activates the PVR/TIGIT pathway by promoting PVR transcription, thereby inhibiting tissue-resident memory T cell activation (161). In breast cancer, the CD155-CD226/TIGIT/CD96 immune checkpoint complex is expressed on both tumor cells and tumor-infiltrating lymphocytes, demonstrating diverse prognostic implications (162). Therapeutic strategies targeting TIGIT show considerable promise across multiple malignancies: in pancreatic ductal adenocarcinoma, the bispecific antibody chi2B5×4F11 targeting both TIGIT and CDCP1 effectively restores T/NK cell function (163); in non-small cell lung cancer, the anti-TIGIT monoclonal antibody Tiragolumab combined with PD-1/PD-L1 inhibitors significantly improves objective response rates (164); and in colorectal cancer, the TIGIT+PD-1+CXCL13+ CD8+ T cell population has been identified as a potential prognostic biomarker (165). Notably, combined blockade of PD-1 and TIGIT promotes CD226-driven clonal expansion of tumor-specific CD8+ T cells while preventing their exhaustion (166). Furthermore, the combination of Serplulimab with anti-TIGIT/LAG-3 inhibitors demonstrates superior antitumor efficacy compared to other PD-1 inhibitors (167). Collectively, these findings indicate that targeting TIGIT and its associated pathways represents a promising therapeutic strategy for various cancer types.

### 4.2 Hematologic malignancies

In hematological malignancies, the mechanisms underlying the PD-1/PD-L1 pathway are also being progressively elucidated. In classical Hodgkin lymphoma (cHL), PD-L1 gene amplification at 9p24.1 leads to high PD-L1 expression on Reed–Sternberg cells. Nivolumab monotherapy has demonstrated an ORR of 69% and a complete response rate of 16% in relapsed/refractory cHL (168, 169). In B-cell non-Hodgkin lymphomas, PD-L1 structural rearrangements have also been shown to correlate with elevated protein expression strongly (157). In diffuse large B-cell lymphoma (DLBCL), PD-L1 translocations drive aberrant overexpression, while miR-155 enhances PD-L1 levels by targeting its 3′ untranslated region, thereby promoting immune evasion (170, 171). In acute T-lymphoblastic leukemia (T-ALL), MYC directly activates the PD-L1 promoter, inducing its expression, suggesting that MYC is a key regulator of immune escape. Inhibiting MYC may restore anti-tumor immune responses (172).

Upregulation of LAG-3 expression has been observed in TILsor tumor cells from patients with chronic lymphocytic leukemia (CLL), AML, Hodgkin lymphoma, and DLBCL, and is closely associated with poor prognosis (173–176). In the peripheral blood and bone marrow of CLL patients, LAG-3^+^PD-1^+^ T cells are significantly increased, suggesting a potential role in the development of immune exhaustion (173). Blockade of LAG-3 markedly enhances the activation capacity of peripheral T cells from CLL patients and synergistically improves anti-leukemia effects when combined with PD-1 antibodies (174, 177). The bispecific antibody tebotelimab, targeting both LAG-3 and PD-1, has shown therapeutic efficacy in relapsed/refractory hematologic malignancies such as DLBCL, accompanied by upregulation of CD8^+^ T cell activation markers, further confirming its immunoenhancing potential (178).

TIM-3 has been found to be widely expressed in immune cells and the bone marrow microenvironment in AML and high-risk myelodysplastic syndromes, where it contributes to immune suppression and the maintenance of leukemia stem cells. Sabatolimab (MBG453), a humanized IgG4 monoclonal antibody targeting TIM-3, is the first agent of its class to enter clinical evaluation. It exhibits high-affinity blockade of TIM-3 interactions with ligands such as Galectin-9 and phosphatidylserine, demonstrating dual immunomodulatory and microenvironment-regulating effects (179). In the Phase I STIMULUS study (NCT03066648), Sabatolimab combined with hypomethylating agents achieved an ORR of 56.9% in patients with HR/vHR-MDS, with a median duration of response of 17.1 months (180).

In hematologic malignancies, aberrant activation of the TIGIT pathway has been identified as a key mechanism mediating immune escape. A series of studies demonstrate that in AML, TIGIT contributes to disease progression through multiple mechanisms: not only is TIGIT broadly overexpressed in the AML microenvironment (93), but its expression patterns also show significant correlation with clinical outcomes. Specifically, a high frequency of bone marrow TIGIT+PD1+ CD8+ T cells is independently associated with T cell exhaustion and inferior relapse-free survival (181). Concurrently, expansion of the PD-1+TIGIT+CD226- CD8+ T cell subset is significantly linked to T cell dysfunction and poor prognosis (182). In core-binding factor AML, a distinct subtype, TIGIT-positive NK cells similarly exhibit impaired cytotoxic function, and their elevated frequency predicts poor relapse-free survival (183). Notably, an analogous immune evasion mechanism is observed in multiple myeloma, where malignant plasma cells upregulate TIGIT, leading to functional impairment and cell death of bone marrow ILC2s, while peripheral blood ILC2s retain their cytotoxic capacity (184). These collective findings highlight the promising therapeutic potential of targeting the TIGIT pathway. Of particular importance, a pivotal study confirmed that the anti-TIGIT monoclonal antibody tiragolumab, in combination with the hypomethylating agent azacitidine, exerts synergistic anti-tumor effects (93), providing a direct and promising new avenue for immunotherapy in hematologic malignancies.

## 5 Conclusion and outlook

The discovery and clinical application of immune checkpoint molecules have significantly advanced the field of cancer immunotherapy. Inhibitory checkpoints such as CTLA-4 and PD-1/PD-L1 have been widely applied in various solid tumors and hematologic malignancies, markedly improving survival outcomes in a subset of patients. Meanwhile, research on costimulatory molecules including CD28, ICOS, and 4-1BB has opened new avenues for enhancing T cell function and reversing immune suppression. In recent years, emerging checkpoints such as LAG-3 and TIM-3 have entered clinical investigation, and multi-target combinatorial strategies are becoming a critical breakthrough for improving therapeutic response rates and durability.

Nevertheless, current immunotherapy still faces several challenges, including interpatient variability in therapeutic efficacy, the frequent emergence of resistance, and immune-related adverse events. Future research should focus on elucidating the precise regulatory mechanisms of immune checkpoint molecules and delineating their functional characteristics within distinct tumor microenvironments. Concurrently, efforts should be directed toward the development of novel antibodies, small-molecule drugs, and nanomedicines, as well as the establishment of accurate and efficient biomarker systems, with the ultimate goal of achieving more personalized and targeted therapeutic strategies.

Notably, the application of immunotherapy is gradually extending to earlier stages of disease, including postoperative adjuvant therapy, intervention in precancerous lesions, and the control of micrometastases. Moreover, combination strategies involving chemotherapy, targeted therapy, and radiotherapy have demonstrated strong synergistic and complementary effects. With continued advances in mechanistic research and therapeutic technologies, immune checkpoint–targeted therapies are expected to play an increasingly pivotal role in cancer treatment, offering more durable survival benefits—and even the possibility of a cure—for a broader population of patients.

## Glossary

AML: acute myeloid leukemia
APCs: antigen-presenting cells
BC: breast cancer
CAR-T: chimeric antigen receptor T-cell
CHL: classical Hodgkin lymphoma
CLL: lymphocytic leukemia
CTLA-4: T-lymphocyte-associated protein 4
CRC: Colorectal Cancer
DCs: dendritic cells
DLBCL: diffuse large B-cell lymphoma
EMT: epithelial–mesenchymal transition
EC: Endometrial Cancer
HCC: hepatocellular carcinoma
ESCC: Esophageal Squamous Cell Carcinoma
ES-SCLC: Extensive-Stage Small Cell Lung Cancer
HNSCC: Head and Neck Squamous Cell Carcinoma
ICB: immune checkpoint blockade
ICOS: Inducible T-cell Co-Stimulator
IL-2: interleukin-2
LAG-3: lymphocyte activation gene-3
LC: lung cancer
MM: melanoma
MCC: Merkel Cell Carcinoma
NK: natural killer
NSCLC: non-small cell lung cancer
NSCLC: non–small cell lung cancer
ORR: objective response rate
OC: Ovarian Cancer
OS: overall survival
PD-1: programmed cell death protein 1
PDCD1: Programmed Cell Death 1
PD-L1: programmed death-ligand 1
PFS: progression-free survival
RCC: Renal Cell Carcinoma
T-ALL: T-lymphoblastic leukemia
TMB-high: Tumor Mutational Burden-High
TCR: T cell receptor
Tfh: follicular helper T cells
TILs: tumor-infiltrating lymphocytes
TIM-3: T cell immunoglobulin and mucin-domain containing-3
TNBC: triple-negative breast cancer
Tregs: regulatory T cells
TRIM: T cell receptor–interacting molecule
TIGIT: T cell immunoreceptor with immunoglobulin and ITIM domains
UC: Urothelial Carcinoma
VISTA: V-domain Ig suppressor of T cell activation
4-1BB: Tumor necrosis factor receptor superfamily member 9
